# Problems and Promises of Introducing the Magnetic Resonance Imaging Linear Accelerator Into Routine Care: The Case of Prostate Cancer

**DOI:** 10.3389/fonc.2020.01741

**Published:** 2020-09-02

**Authors:** Charisma Hehakaya, Jochem R. Van der Voort van Zyp, Jan J. W. Lagendijk, Diederick E. Grobbee, Helena M. Verkooijen, Ellen H. M. Moors

**Affiliations:** ^1^Division of Imaging & Oncology, University Medical Center Utrecht, Utrecht, Netherlands; ^2^Julius Center for Health Sciences and Primary Care, University Medical Center Utrecht, Utrecht, Netherlands; ^3^Copernicus Institute of Sustainable Development, Utrecht University, Utrecht, Netherlands

**Keywords:** cancer care, healthcare management, image-guided radiotherapy, implementation, MRI-guided radiotherapy, MR-Linac, prostate cancer, qualitative research

## Abstract

The new radiotherapy high field, 1.5 Tesla MRI-guided linear accelerator (MR-Linac) is being clinically introduced. Sensing and evaluating opportunities and barriers at an early stage will facilitate its eventual scale-up. This study investigates the opportunities and barriers to the implementation of MR-Linac into prostate cancer care based on 43 semi-structured interviews with Dutch oncology care professionals, hospital and division directors, patients, payers and industry. The analysis was guided by the Non-adoption, Abandonment, Scale-up, Spread, and Sustainability framework of new medical technologies and services. Opportunities included: the acquirement of (1) advanced MRI-guided radiotherapy technology with (2) the potential for improved patient outcomes and (3) economic benefits, as well as (4) professional development and (5) a higher hospital quality profile. Barriers included: (1) technical complexities, (2) substantial staffing and structural investments, (3) the current lack of empirical evidence of clinical benefits, (4) professional silos, and (5) the presence of patient referral patterns. While our study confirms the expected technical and clinical prospects from the literature, it also reveals economic, organizational, and socio-political challenges.

## Introduction

The implementation of medical technology and services usually involves individual, organizational and environmental factors (1–4). All three are relevant to the introduction of MRI-guided linear accelerator (MR-Linac) systems: the 0.35 Tesla ViewRay MRIdian system and the 1.5 Tesla Elekta Unity system (5, 6). Yet, their introduction into routine oncology care has mainly been reported from a technical and clinical perspective (7–10). In this study we will focus on the Unity MR-Linac, recently developed by the University Medical Center Utrecht (Utrecht, The Netherlands) in collaboration with Elekta AB (Stockholm, Sweden) and Philips (Best, The Netherlands). This technology integrates a 1.5 Tesla MR-imaging scanner with a radiotherapy linear accelerator (9, 11–15). This enables online adaptive radiotherapy delivery and diagnostic quality imaging simultaneously that allows the visualization of tumor and surrounding organs before, during and after treatment (9, 12, 16–19), with potentially higher treatment accuracy, the sparing of healthy tissue and the possibility of hypofractionation (providing the total dose in fewer treatment sessions). These features are expected to deliver real health benefits for patients including better tumor control, fewer side effects, and a shorter treatment course (17, 20–22). Since MR-Linac’s CE approval in June 2018 and FDA approval in December 2018, the technology has been installed in institutions worldwide (23, 24).

Despite the promising clinical and technological prospects, challenges remain. The use of MR-Linac requires high capital investments in equipment, logistics, quality assurance and complementary training (7), and evidence of superior patient outcomes (8). Implementing technical developments in cancer treatment may disrupt standard treatment practices, which call for financial considerations, collective learning, and organizational renewal (25, 26). Collective learning and organizational renewal can be hampered by hospital autonomy and by cultures of secrecy within specialties (2, 3, 27, 28). These are potential bottlenecks that are seldom investigated in radiotherapy centers (29). While some attention has been given to potential implementation challenges, these aspects need untangling and a clearer understanding in order to maximize benefits and to avoid setbacks for patients and care givers (8, 17, 19, 25, 30).

This study aims to identify the opportunities and barriers for successful implementation of MR-Linac into prostate cancer care. The choice for focusing on prostate cancer is based on the following reasons. First, online adaptive radiotherapy is most likely to be of benefit for tumors that move between and during treatment (13, 31), as is the case in prostate cancer (18, 32). Prostate cancer, the most common cancer in men worldwide, does have high survival rates (33, 34). However, current treatments may interfere with quality of life: external beam radiotherapy, brachytherapy, and even minimally invasive robotic procedures, cause adverse effects such as erectile dysfunction and urinary incontinence (33–35). MR-Linac minimizes uncertainties about the actual tumor’s location, shape and the surrounding organs at risk, which may reduce adverse effects and in turn improve a patient’s quality of life (9, 11). Second, clinical interest in MRI-guided radiotherapy in prostate cancer management has been increasing in recent years (10, 17, 36), and understanding the dynamics of its implementation is timely.

## Materials and Methods

### Design

We conducted a qualitative study including semi-structured interviews, an approach most appropriated to make sensitive issues and attitudes, opinions and experiences of individuals explicit (37). We used the Non-adoption, Abandonment, Scale-up, Spread and Sustainability (NASSS) framework of new healthcare technologies and services which is designed to explore determinants of success and failure of technology adoption in healthcare organizations. The NASSS framework considers seven domains: the condition or clinical indication, the technology to be implemented, the value proposition, the adopter system (patient, technology user and other staff), the organization, the wider institutional and social context, and organizational resilience and technology development over time (38).

### Recruitment

Respondents were recruited through purposive and snowball sampling using recommendations from initial respondents. We wanted to obtain a convenience sample of compelling roles among the populations of interest. Because implementing medical technologies and services requires a comprehensive multilevel consideration of individual, organizational and environmental influences (1–4), we attempt to select respondents at each of these levels of influence and based on their expertise. Therefore, we adopted a number of selection criteria:

1. Working at a hospital offering MR-Linac treatment; or
2. Providing other prostate cancer treatments (e.g., external beam radiotherapy, low- or high-dose-rate brachytherapy, proton beam therapy, robotic surgery, radiosurgery); or
3. Management experience relevant to the implementation and insurance coverage of new medical technologies or services; or
4. Stakeholders outside the hospital (e.g., patients, care insurers, manufacturing industry).

We included physicists, radiation oncologists, radiotherapy technologists and ICT staff, currently practicing MR-Linac (9). We also interviewed urologists (the referring physician in the Netherlands), radiologists and nuclear medicine physicians. Further, we included hospital directors, division and insurance managers. We included respondents from different hospitals, to limit selection bias. At the time of writing this article, only two hospitals offer Unity MR-Linac treatment in the Netherlands. This country is a suitable context, considering that it has been the first nation in which this technology has been introduced. At the contextual level, we included the perspectives of patients, care insurers and the executives of industries that hold MR-Linac’s intellectual property rights. Our respondents, except those in industries, are located in Netherlands.

### Data Collection

The research objective was explained in the invitation and at the start of each interview. The questionnaire is based on the interview questions of the NASSS framework and the first interviews (see Supplementary Appendix A). It included open-ended questions to explore each respondent’s experience with and views on MR-Linac for prostate cancer treatment, including implementation opportunities and barriers. All interviews were conducted by one trained researcher and lasted approximately 45 minutes until saturation occurred and no new information appeared in the data. Interviews were conducted in person (*N* = 35), by phone (*N* = 5), or by Skype (*N* = 3). All interviews were audio-recorded with the consent of the respondents and transcribed. Each respondent validated their transcript. Audio recordings and transcript of interviews are confidential and therefore not publicly available.

### Data Analysis

Interview transcripts were analyzed in NVivo software. We first applied open coding based on the research objective. We then applied axial coding, systematically identifying areas of interest based on the NASSS framework. This iterative step involved repetitions aimed at revising primary codes. We triangulated responses across different respondents and subsequently identified the opportunities and barriers. Resulted codes were validated by a second reviewer. We regularly discussed whether the empirical data matched the NASSS framework, ensuring that results were correctly classified within their domain. To include variation in findings and increase construct validity, we interviewed more than one person per profession and also considered perspectives from various hospitals.

## Results

**FIGURE 1:**
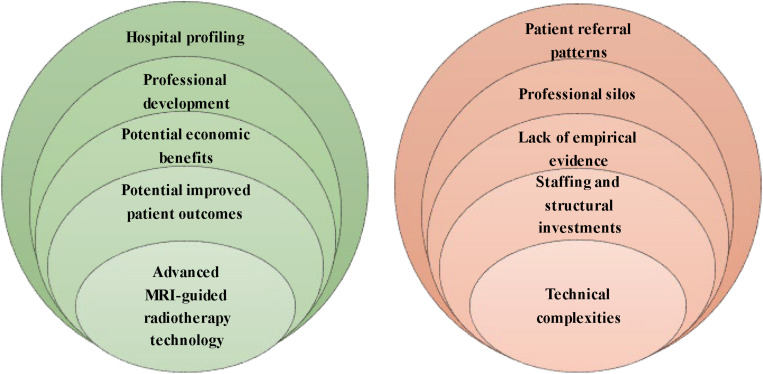
Overview of opportunities and barriers.

We conducted 43 interviews with professionals in MRI-guided radiotherapy as well as other prostate cancer treatments, hospital and department directors, insurance commissioners, and external stakeholders between November 2018 and March 2019 (see Table 1). Hospital respondents work in four academic and three non-academic Dutch hospitals, of which two hospitals installed Unity MR-Linac and one hospital ViewRay MRIdian. Five opportunities and five barriers to the implementation of MR-Linac have been identified (see Figure 1). We first present the opportunities, followed by the barriers according to the frequency stated by the respondents.

**TABLE 1:** Overview of roles and affiliations of respondents.

Respondent	Position	Seniority	Affiliation	Selection criteria	Additional roles	Method	Duration (min)
R1	Computer scientist	Senior	AMC1	1	Research on MR-Linac	In Person	43
R2	Manager Imaging & Oncology dep.	Senior	AMC1	3		In Person	47
R3	Head of Imaging & Oncology dep.	Full Professor	AMC1	3	Research on functional imaging	In Person	44
R4	Insurance commissioner	Senior	AMC1	3		In Person	25
R5	Insurance commissioner	Senior	AMC1	3		In Person	31
R6	Member Board of Directors	Full Professor	AMC1	3	Research on Open Science	In Person	30
R7	Member Board of Directors	Senior	AMC1	3		In Person	44
R8	Member Board of Directors	Full Professor	MC1	3	Radiologist	In Person	45
R9	Nuclear medicine physician	Full Professor	AMC1	2	Board Member National Education Committee Nuclear Medicine	In Person	43
R10	Nuclear medicine physician	Senior	AMC2	2		In Person	41
R11	Radiologist	Senior	AMC1	2		In Person	42
R12	Radiologist	Senior	AMC1	2		In Person	40
R13	Radiation oncologist	Senior	AMC1	1,2	Research on MR-Linac	In Person	37
R14	Radiation oncologist	Senior	AMC1	1,2	Research on MR-Linac	In Person	41
R15	Radiation oncologist	Senior	AMC1	1,2	Research on MR-Linac	In Person	43
R16	Radiation oncologist	Senior	AMC1	1,2	Research on MR-Linac	In Person	45
R17	Radiation oncologist	Full Professor	AMC2	2,3	Head of Radiation Oncology department, Research on MR-Linac, Board member European Society Radiotherapy & Oncology	In Person	46
R18	Radiation oncologist	Senior	AMC3	2	Head of Radiation Oncology department	Telephone	39
R19	Radiation oncologist	Senior	AMC4	2		In Person	45
R20	Radiation oncologist	Full Professor	AMC4	2,3		In Person	48
R21	Radiation oncologist	Senior	MC2	2	Research on MR-Linac	In Person	41
R22	Radiotherapy technologist	Senior	AMC1	1,2	Research on MR-Linac	In Person	43
R23	Radiotherapy technologist	Senior	AMC1	1,2	Research on MR-Linac	In Person	39
R24	Radiotherapy technologist	Junior	AMC1	1,2		In Person	42
R25	Radiotherapy technologist	Senior	MC2	1,2		Telephone	46
R26	Radiotherapy technologist	Senior	AMC3	2		In Person	44
R27	Physicist	Full Professor	AMC1	1,2,3	Research on MR-Linac	In Person	53
R28	Physicist	Senior	AMC1	1,2	Research on MR-Linac	In Person	41
R29	Physicist	Full Professor	AMC1	1,2	Research on MR-Linac	In Person	45
R30	Physicist	Senior	AMC1	1,2	Research on MR-Linac	In Person	44
R31	Physicist	Senior	AMC2	1,2	Research on MR-Linac	Telephone	38
R32	Physicist	Full Professor	AMC2	2,3	Manager Radiation Oncology department	In Person	41
R33	Physicist	Full Professor	AMC3	2		Online	39
R34	Physicist	Full Professor	MC2	1,2	Research on MR-Linac	In Person	43
R35	Physicist	Senior	MC2	1,2	Research on MR-Linac	In Person	47
R36	Urologist	Senior	AMC1	2		In Person	31
R37	Urologist	Senior	MC3	2		Telephone	29
R38	Urologist	Senior	AMC4	2	Head of Urology department	Telephone	40
R39	Patient representative		National patient organization	4		Telephone	31
R40	Healthcare insurer	Senior	Insurance company 1	4	Radiologist	Telephone	38
R41	Healthcare insurer	Senior	Insurance company 2	4		Telephone	42
R42	Managing Director	Senior	Manufacturing company	4		Online	46
R43	Managing Director	Senior	Manufacturing company	4		Online	33

*AMC, academic medical center; MC, (non-academic) medical center.*

### Opportunities

**FIGURE 2:**
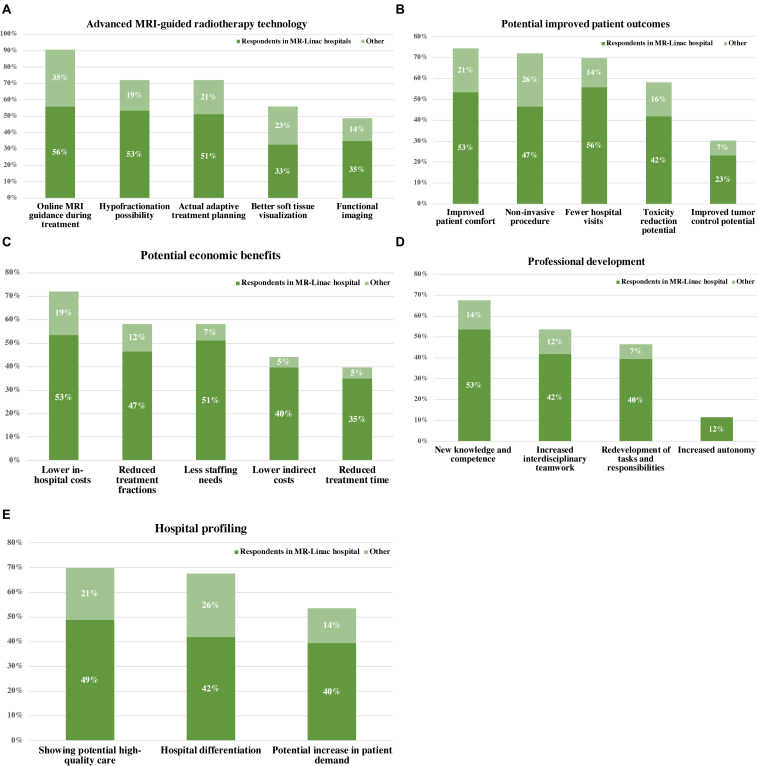
Percentages of the interview cohort who discussed opportunities to the implementation of MR-Linac into prostate cancer care, by main theme and subtheme. **(A)** Advanced MRI-guided radiotherapy technology, **(B)** potential improved patient outcomes, **(C)** potential economic benefits, **(D)** professional development, and **(E)** hospital profiling.

Our respondents revealed five opportunities to MR-Linac implementation for prostate cancer: (1) advanced MRI-guided radiotherapy technology, (2) potential improvement in patient outcomes, (3) potential economic benefits, (4) professional development, and (5) hospital profiling. Figure 2 shows the percentages of the interview cohort who discussed the opportunities, by main theme and subtheme. Supplementary Appendix B provides an overview of the respondents referencing opportunities.

#### Advanced MRI-Guided Radiotherapy Technology

Given the increasing demand in radiotherapy for advanced image-guidance and adaptive treatments subsequently, the use of MRI during radiotherapy is perceived as an inevitable follow-on advancement in this field. The opportunity of real-time diagnostic-strength 1.5 Tesla MRI-imaging that enables better soft tissue visualization; daily on-table adaptation to anatomical changes; actual adaptive treatment planning; hypofractionation and evaluation of tumor response during the course of radiotherapy. Further, actual anatomical and functional information of the prostate tumor and greater confidence in avoiding organs at risk during treatment is perceived as very promising, allowing more accurate, targeted treatment and avoiding radiation of healthy tissue. According to current technology users, these prospects promise new treatment avenues in radiation oncology as well as in related medical disciplines.

#### Potential Improved Patient Outcomes

Prostate cancer is a well-characterized disease with effective treatment modalities. However, the potential adverse effects of present treatments are substantial and can interfere with the patient’s quality of life; this remains a key target in present treatment development. Radiotherapy practitioners and members of hospital management expect MR-Linac to solve this issue and to yield improved patient outcomes. The majority of respondents mentioned improved patient comfort as main benefit resulting from: (1) possibly fewer adverse effects, (2) possibly improved tumor control, (3) the non-invasive procedure; the implantation of gold fiducial markers within the prostate is no longer necessary for position verification, and (4) hypofractionation allows prostate cancer treatment in fewer hospital visits and may shorten waiting lists. For example, the current standard is to give prostate radiotherapy in 20 fractions and hypofractionation has the potential to perform to allow completion of the entire treatment in only 2–5 times.

#### Potential Economic Benefits

In view of the current, unsustainable growth in medical expenditures, the present-day value of new treatments will require an improvement in both treatment quality and cost reduction. According several radiotherapy professionals MR-Linac may offer quality and efficiency gains. First, both the preparation of the treatment plan as well as the execution takes place on the same device. Second, digital developments, such as deep learning, may allow operational benefits: automation processes during treatment (e.g., automatic contouring of tumor and organs at risk) to reduce staffing needs and waiting times. Ultimately, improved efficiency, together with fewer treatment sessions, fewer hospital visits, potentially fewer adverse effects and lower direct in-hospital costs such as anesthesia provision or indirect care costs (e.g., treatment of adverse effects and transport costs) can reduce overall costs.

#### Professional Development

Implementing MR-Linac allows room for professional development and multidisciplinary learning. First, users experience an increased communication and collaboration across radiation oncology and imaging specialties (e.g., for the development of scanning protocols on MR-Linac). In most hospitals diagnostics and treatment are performed by different groups and the interaction between them is therefore limited. Second, the required knowledge of both MR-imaging and radiotherapy integrates different competences and expertise. As consequence, MR-Linac users may be attracted by the development and use of new knowledge and competences and the redevelopment of tasks and responsibilities. Third, radiotherapy technologists also reported their potential increased autonomy and involvement in decisions. They would have more responsibility like the maintenance of MRI protocols and active safeguarding of radiation requirements for target volume and organs at risk. The empowerment of employees fosters a better workplace culture.

#### Hospital Profiling

The implementation of MR-Linac also offers hospitals a way to profile themselves as innovative; providing potentially high-quality care. They also expect that hospitals implement MR-Linac to keep up with recent developments in radiation oncology and attract patients accordingly. According to the patient representative and several professionals, the target population is generally aware of the treatment modalities and prefers MRI-guided treatment. This could increase patient referral to the radiotherapy department and related medical specialties. Implementing MR-Linac could therefore provide hospitals a competitive advantage.

### Barriers

**FIGURE 3:**
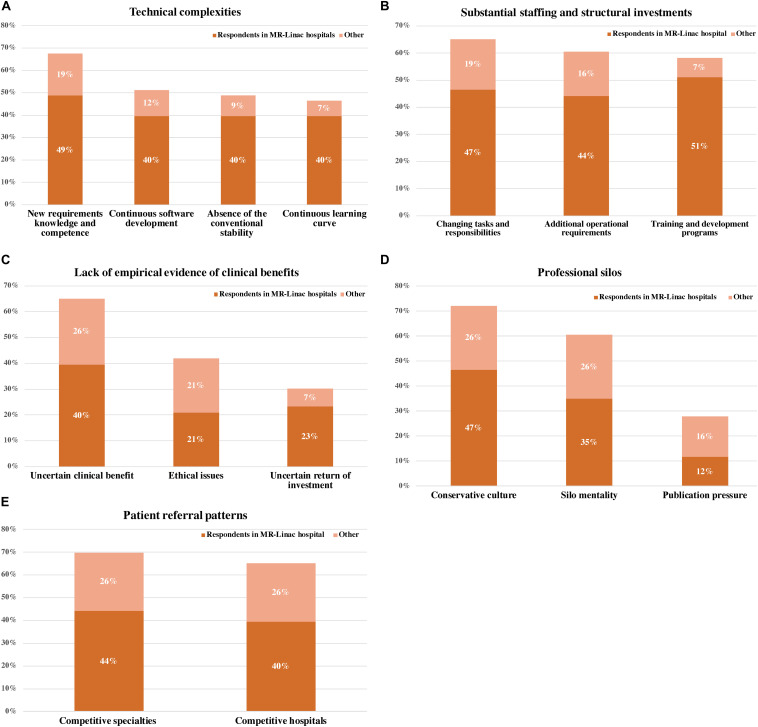
Percentages of the interview cohort who discussed barriers to the implementation of MR-Linac into prostate cancer care, by main theme and subtheme. **(A)** Technical complexities, **(B)** substantial staffing and structural investments, **(C)** Lack of empirical evidence of clinical benefits, **(D)** professional silos, and **(E)** patient referral patterns.

Our respondents revealed five main barriers to MR-Linac implementation for prostate cancer: (1) technical complexities, (2) staffing and structural investments, (3) the lack of empirical evidence of clinical benefits, (4) professional silos, and (5) the presence of patient referral patterns. Figure 3 shows the percentages of the interview cohort who discussed the barriers, by main theme and subtheme. Supplementary Appendix C provides an overview of the respondents referencing barriers.

#### Technical Complexities

The involvement of MRI in radiotherapy is expected to transform current radiation oncology practice in terms of target identification, tumor response assessment, treatment planning and delivery, quality assurance and staffing. MR-Linac’s ultimate impact on the current radiation oncology development is not yet known considering its continuous development, which largely depends on software upgrades rather than hardware upgrades. The technology’s output is vulnerable to the interpretations of individual practitioners and may associate with inter- and intra-variability in treatment procedure, which in turn could affect clinical outcomes. Hence, the absence of the conventional security of the traditional linear accelerator necessitates the presence of experienced staff. This, together with continuing software developments, requires users to anticipate an ongoing learning curve.

In practice, MR-Linac’s value is limited by software challenges in real-time tumor tracking during radiation. One treatment session is relatively also longer compared to conventional external beam radiotherapy, and this longer treatment duration could be a potential barrier for the patient. Each treatment lasts approximately 45 min, which is three to four times longer than conventional external beam radiotherapy (22).

#### Substantial Staffing and Structural Investments

The required MRI competence, knowledge and the need for on-the-spot decision making were at the same time also seen as a challenge. For example, a radiation oncologist reported that brachytherapy practitioners are more used in making decisions on the spot than those involved in conventional external beam radiotherapy only. Adequate training programs are therefore a prerequisite to ensure that MR-Linac is used effectively and that MRI is safe for both patients and users. Further, several respondents also mentioned the need to expand the responsibilities of radiotherapy technologists to reduce the presence of the radiation oncologist and physicist during treatment and staffing costs subsequently. Although radiation technologists could bear more responsibility, other concerns are their limited availability and that existing Dutch policy does not allow therapists to approve treatment plans.

Another perceived barrier is the substantial structural investments required: today’s radiotherapy centers often lack the needed combination of MR-imaging and radiation facilities. To illustrate, a single MR-Linac costs 10 million euros without the requisite infrastructure, such as MRI compatibility, MRI safety, clinical workflow and its accompanying software development, quality assurance and the development of protocols, roles and responsibilities. Early adopters are therefore well financed medical research centers with MR-imaging expertise and facilities. Accordingly, MR-Linac reflects the trend that cancer care is increasingly centralized (39).

#### Lack of Empirical Evidence of Clinical Benefits

Despite promising theoretical benefits, clinical value remains undocumented and the patient categories that will most benefit remain unclear. For present prostate radiotherapy, there is some room for improvement in terms of adverse effects and patient comfort. However, some respondents doubted the actual reduction in toxicity and clinical added-value. Also, respondents doubted whether hypofractionation would actually compensate for the increased cost because of more expensive technology, increased treatment time per fraction and organizational investments (e.g. the requirement of more highly trained staff). Further, few respondents questioned the clinical added-value of MR-Linac compared to ViewRay MRIdian as well as other potential emerging techniques in prostate cancer treatment (e.g., CT-based adaptive radiotherapy).

The present lack of empirical evidence also explains MR-Linac’s lack of insurance coverage. Consequently, this can hamper real savings for hospitals and care insurers, as the potential reduction in treatment costs cannot be achieved. Further, the provision of treatment with unproven efficacy and safety to the patient may also lead to ethical discussions. High-quality randomized control trials are imperative to compare the value of MR-Linac with alternative treatments: preferably with comparable outcomes across different centers. A multi-center clinical evaluation would also hasten the recruitment of patients needed. Paradoxically, our respondents reported the lack of clinical evidence hindering successful implementation, while also mentioning the need to install the technology in a clinical environment for empirical evaluation.

#### Professional Silos

Amongst the redevelopment of tasks and responsibilities, practicing MR-Linac can threaten users’ professional identity. Several radiotherapy professionals reported the potential conservative behavior and resistance as response to delegate tasks and change daily practice. Another perceived barrier is the publicity pressure exerted upon medical research centers which may hamper knowledge exchange and open communication about MR-Linac between hospitals. The political climate can hinder effective multicenter collaboration within and across hospitals, and the technology’s further development. These challenges relate to the silo mentality and conservative culture that often prevails in hospitals.

#### Patient Referral Patterns

Finally, introducing MR-Linac into routine care could raise patient referral discussions among specialties where patient demand may be compromised. The relationship between radiation oncology and surgery can be complementary, but also be competitive (26). In the Dutch prostate cancer care, urologists play an important role in patient access to MR-Linac as they discuss the treatment modalities with the patient. Likewise, radiotherapy centers offering MR-Linac may also be a perceived threat to hospitals that do not offer this technology, and hence would resist patient referral to this treatment.

## Discussion and Implications for Practice and Future Research

Our findings help radiation oncology departments determining focus areas in their strategy for successful MR-Linac implementation into prostate cancer care. Consistent with prior research, MR-Linac users expect to benefit from advanced MRI-guided radiation technology with online adaptive treatment and response assessment that may potentially improve patient outcomes and identify new treatment opportunities (7, 8, 10, 19, 40). The possibility of prostate hypofractionation promises improved treatment and economic benefits (17, 20, 41–43). Users boost their hospital profile and professional development, irrespective of radiation oncologists, technologists and physicists (7, 8, 25, 44, 45). Our study also confirms the need to generate clinical evidence, while dealing with technical complexities and substantial staffing and structural investments (7). However, simply addressing these barriers is not enough: successful implementation also raises economic, organizational and socio-political concerns embedded in the presence of patient referral patterns and professional silos. These concerns are understudied in the current efforts on MR-Linac implementation into routine prostate cancer care.

Many respondents perceive MR-Linac as a complex innovation with a high implementation burden: its multidisciplinary nature disrupts the traditional barrier between radiation oncology and diagnostic radiology (7, 10, 26) which practically justifies all barriers. The involvement of MRI in radiation oncology transforms current practices either within and outside the radiation oncology department (7). Users are clearly concerned with substantial structural and staffing investments, established determinants in new technology and service implementation in healthcare (21, 46). The substantial investments are also explained by the technical complexities inherent in MR-Linac. Further, our respondents have identified concerns about software deficiencies and the relative longer treatment fractions. Technological development should focus on improving workflow and the automation of both imaging and treatment (12, 36, 47).

MR-Linac’s technical character has a major impact on staffing roles, which can lead to both efficiency improvements and professional identity threats. The ongoing technology development together with the acquirement of new skills (e.g., MRI competence, on-the-spot decision-making) illustrate the need for users to anticipate new learnings and responsibilities. However, the transformation of existing staff roles is not easy and is perceived as more than just learning how to use a new technology. This would require acceptance of changes in professional identity and autonomy as well as increased communication across disciplinary boundaries. Moreover, current staffing policies in radiation oncology impede the reallocation of responsibilities for radiotherapy technologists. Technology users should therefore invest in workplace training and development with supporting staffing policies. Radiotherapy education will have to change in order to prepare physicists, radiation oncologists and technologists on the technical developments of MR-Linac. Further impact studies can focus on the professional development of users and the right staff policy to ensure a sustainable use of MR-Linac.

The reallocation of staffing is made more difficult by the presence of professional silos. Interestingly, in prostate cancer and cancer treatment in general, communication and cooperation between different disciplines tangled have already been proposed as prerequisites in effective cancer care (48–51), however, these features are still being raised as potential hindrances in MR-Linac implementation. Professional silos can be expressed by the presence of specialisms and related conservative behavior, a common challenging determinant in changing existing practices in hospitals (27), which also applies here. This also impedes the smooth collaboration and integration of diagnostic imaging and radiotherapy.

Another barrier is the likely presence of patient referral patterns. Safeguarding patients’ access to MR-Linac requires participation of radiotherapy professionals as well as referring physicians (the urologist in the context of Netherlands). Ultimately, successful implementation would therefore require active support and participation from hospital executives, and alignment between departments (radiotherapy and urology. The required communication and collaboration strengthen horizontal connections between different disciplines (e.g., radiation oncology and imaging), but also vertical connections inside (e.g., between radiotherapy technologist and radiation oncologist) and outside the radiotherapy department.

Future efforts should generate clinical evidence to prove expectations and justify return on investment concerns, an indispensable determinant in technology implementation which has been given greater emphasis in the new European Medical Device Regulation (46, 52). Evaluation of an evolving technology such as MR-Linac is very difficult (53). Therefore, the international Unity MR-Linac consortium (9) has set up a prospective registry to include patients treated on MR-Linac in seven large institutions (MOMENTUM registry). Here, patients provide informed consent for the use of their technical (imaging) and clinical data for academic and clinical research as well as response assessment. Costs and quality of life data will be collected as well, to identify cost-effective MR-Linac treatment strategies compared to alternative treatments. This is particularly useful in the field of prostate cancer, where many treatment modalities with comparable outcomes, but with different costs are available (54). Further MR-Linac impact studies can also provide insights into its effects on prostate cancer treatment allocation and hospital infrastructure.

The lack of clinical evidence also causes gaps in insurance coverage. This, together with the substantial investments, creates a high implementation burden and uncertainty for potential MR-Linac users and payers. Interestingly, this has not prevented radiation oncology departments from implementing the technology. The increasing belief in image-guided technologies without proven results to profile users with state-of-the-art treatments and high quality care also applies to MR-Linac (8, 25). Despite the mutual skepticism among fellow professionals and health insurers about the clinical added-value of MR-Linac, collaboration between them facilitates technology users to meet requirements in treatment evaluation for insurance coverage.

Our study provides the first multifaceted assessment of opportunities and barriers in MR-Linac implementation for prostate cancer including perspectives from professionals, hospital and division directors, patients, payers and industry. The value of qualitative research is to explore phenomena in-depth and to question respondents about their relevant knowledge, opinions and experience. While a more extensive and systematic sampling method would limit selection bias, this study likely captures a significant proportion of the relevant qualitative information. Interviews with early adopters revealed hitherto unanticipated implementation challenges (29). Typical feasibility or cost-effectiveness studies would overlook the potential effects of potential resistance to patient referral, changing practice habits and silo mentality. Whereas this study focuses on prostate cancer, the operational and organizational prospects discussed by respondents are likely to be valid for the implementation of MR-Linac for other tumor indications as well. A comprehensive comparison between MR-Linac systems [e.g., MRIdian of ViewRay (6)] as well as with emerging radiotherapy techniques and present prostate cancer treatments goes beyond the scope of this paper. Finally, generalizability of our findings to other contexts has to be carefully considered. Future efforts can determine how country-specific therapeutic standards, political and social contexts influence MR-Linac’s implementation activities.

## Conclusion

Given the rapid development of MR-Linac, research into factors that stimulate or hamper its local implementation, is needed, as the first step to understand its long-term impact. Our findings define the main opportunities and barriers for successful MR-Linac implementation into routine care. We raise issues that are known in the field but largely overlooked in the current literature on MR-Linac implementation. The discussion of the topics that emerge from the interviews leads to reflection and learning, but also to new connections in MR-Linac implementation and the organization of care. Four fundamental conclusions can be given:

• Successful implementation of MR-Linac not only considers technical and clinical aspects, but also economic, organizational and socio-political challenges.
• MR-Linac implementation is expected to affect present prostate cancer care within and outside the radiation oncology department as well as hospital culture and identity of professionals.
• Involvement of the referring physician is crucial in successfully implementing MR-Linac into routine prostate cancer care.
• Clinical evaluation supported by patients, radiation oncology professionals, referring physicians and payers has to justify MR-Linac’s perceived benefits and substantial investments.

## Data Availability Statement

The datasets presented in this article are not readily available because interview recordings and transcripts are confidential. Requests to access the datasets should be directed to CM, c.hehakaya@umcutrecht.nl.

## Ethics Statement

This study was exempted from ethical approval according to the criteria of Dutch Medical Research involving Human Subjects Act. At the beginning of an interview, each participant was verbally informed about the research objective and interview purpose. All participants provided explicit verbal consent for participation in the interviews and publication of the results.

## Author Contributions

CH conducted the interviews and completed the initial coding of the transcripts, prepared the first draft of this manuscript, and had access to all of the data and final responsibility for the decision to submit for publication. JV, JL, DG, HV, and EM contributed and commented on the draft. All authors contributed to the study design, read and approved the final manuscript, and involved in the analysis of the interview data.

## Conflict of Interest

Several other MR-Linac scientific projects at the Division of Imaging and Oncology of University Medical Center Utrecht have been partly funded Elekta AB (Stockholm, Sweden) and Philips Medical Systems (Best, Netherlands). The authors declare that the research was conducted in the absence of any commercial or financial relationships that could be construed as a potential conflict of interest.

## Supplementary Material

APPENDIX A Questionnaire based on NASSS framework and initial interviews.

APPENDIX B Frequency of respondents who discussed opportunities to the implementation of MR-Linac into prostate cancer care, by main theme and subtheme.

APPENDIX C Frequency of respondents who discussed barriers to the implementation of MR-Linac into prostate cancer care, by main theme and subtheme.

## Abbreviations

MR-Linac: MRI-guided linear accelerator.
