# Supplementary material for: Problems and Promises of Introducing the Magnetic Resonance Imaging Linear Accelerator Into Routine Care: The Case of Prostate Cancer
Source: Front Oncol. 2020 Sep 2;10:1741. doi: 10.3389/fonc.2020.01741 (PMC7493635; doi:10.3389/fonc.2020.01741)
Supplement: APPENDIX A — Questionnaire based on NASSS framework and initial interviews. [file Data_Sheet_1.docx]

Appendix A. Questionnaire based on NASSS framework and initial interviews.

| **Domains and questions**  **from NASSS framework** | **Additional questions**  **based on first interviews** |
| --- | --- |
| 1. Condition | |
| 1. What is the nature of the condition? 2. What are the relevant sociocultural factors? 3. What are the relevant comorbidities? | 1. What are recent trends in the treatment regimen? |
| 1. Technology | |
| 1. What are the key features of the technology? 2. What knowledge and skills are required to use the technology? 3. What is the technology supply model? | 1. What is the current status of the technology development? 2. What are potential condition-specific challenges for the technology? 3. Who owns the intellectual property of the technology? |
| 1. Value proposition | |
| 1. What is the business case for the technology from the supply side? 2. What are its desirability, efficacy, safety and cost-effectiveness from the demand-side? | 1. What are intrinsic and extrinsic motivations to use this technology? 2. What are the requirements to prove the added value of this technology? 3. In which areas does the technology may have an adverse effect? |
| 1. Adopter system | |
| 1. What changes in staff roles, practices and identities are implied? 2. What is expected of the patient, is this achievable and accepted from them? 3. What is assumed about the extended network of lay carers? | 1. Which professions are vital in the introduction of this technology and what are their roles? *[e.g. referring physicians]* 2. To what extent do relevant professions interact with each other when it comes to technology implementation? 3. Which ways improve the interactions between relevant professions when it comes to technology adoption? |
| 1. Organization | |
| 1. What is the organization’s capacity to innovate? 2. How ready is the organization for this technology-supported change? 3. How easy will the adoption and funding decision be? 4. What changes will be needed in team interactions and routines? 5. What work is involved in the implementation and who will do it? | 1. Which intra-organizational discussions will be needed for technology implementation and with whom? 2. Which inter-organizational discussions will be needed for technology implementation and with whom? |
| 1. Wider system | |
| 1. What is the political, economic, regulatory, professional, sociocultural context for technology rollout? | 1. What is the treatment context for technology rollout? |
| 1. Organizational resilience and technology development over time | |
| 1. How much scope is there for adapting and coevolving the technology over time? 2. How resilient is the organization to handling critical events and adapting to unforeseen eventualities? | 1. Which steps can be taken to improve technology implementation? |

Appendix B: Frequency of respondents who discussed opportunities to the implementation of MR-Linac into prostate cancer care, by main theme and subtheme.

| **Opportunities** | **Total** | **Respondent ID, following Table 1** |
| --- | --- | --- |
| **Advanced MRI-guided radiotherapy technology** | | |
| Online MRI guidance during treatment | 39 | 1, 2, 3, 4, 6, 7, 8, 9, 10, 11, 12, 13, 14, 15, 16, 17, 18, 19, 20, 21, 22, 23, 24, 25, 27, 28, 29, 30, 31, 33, 34, 35, 36, 37, 39, 42, 43 |
| Hypofractionation possibility | 31 | 1, 2, 3, 6, 7, 8, 9, 11, 13, 14, 15, 16, 17, 18, 20, 21, 22, 23, 24, 25, 27, 28, 29, 30, 31, 32, 33, 34, 35, 42, 43 |
| Actual adaptive treatment planning | 31 | 1, 2, 3, 4, 8, 9, 11, 13, 14, 15, 16, 17, 18, 19, 20, 21, 22, 23, 24, 25, 27, 28, 29, 30, 31, 33, 34, 35, 39, 42, 43 |
| Better soft tissue visualization | 24 | 9, 11,12, 13, 14, 15, 16, 17, 18, 19, 20, 21, 22, 23, 24, 25, 26, 27, 28, 29, 30, 31, 33, 34, 35, 36, 39, 42, 43 |
| Functional imaging | 21 | 1, 11, 13, 14, 15, 16, 17, 20, 21, 22, 23, 27, 28, 29, 30, 31, 33, 34, 35, 42, 43 |
| **Potential improved patient outcomes** | | |
| Improved patient comfort | 32 | 1, 2, 3, 6, 7, 8, 9, 11, 13, 14, 15, 16, 17, 18, 20, 21, 22, 23, 24, 25, 27, 28, 29, 30, 31, 32, 33, 34, 35, 39, 42, 43 |
| Non-invasive procedure | 31 | 2, 3, 7, 8, 13, 14, 15, 16, 17, 18, 19, 20, 21, 22, 23, 24, 25, 27, 28, 29, 30, 31, 33, 34, 35, 36, 37, 38, 39, 42, 43 |
| Fewer hospital visits | 30 | 1, 2, 3, 4, 6, 7, 8, 11, 13, 14, 15, 16, 17, 20, 21, 22, 23, 24, 25, 27, 28, 29, 30, 31, 32, 33, 34, 35, 42, 43 |
| Toxicity reduction potential | 25 | 2, 3, 6, 7, 8, 13, 14, 16, 17, 20, 21, 22, 23, 25, 27, 28, 29, 31, 32, 33, 34, 35, 39, 42, 43 |
| Improved tumor control potential | 13 | 6, 7, 13, 16, 17, 21, 27, 28, 29, 34, 35, 39, 42, 43 |
| **Potential economic benefits** | | |
| Lower in-hospital costs | 31 | 2, 3, 4, 6, 7, 8, 9, 11, 13, 14, 15, 16, 17, 18, 20, 21, 22, 23, 24, 25, 27, 28, 29, 30, 31, 32, 33, 34, 35, 42, 43 |
| Reduced treatment fractions | 25 | 1, 2, 3, 4, 6, 13, 14, 15, 16, 17, 18, 20, 21, 22, 23, 27, 28, 29, 30, 31, 33, 34, 35, 42, 43 |
| Less staffing needs | 25 | 2, 3, 4, 6, 7, 13, 14, 15, 16, 17, 21, 22, 23, 24, 25, 27, 28, 29, 30, 31, 33, 34, 35, 42, 43 |
| Lower indirect costs | 19 | 2, 3, 6, 13, 14, 15, 16, 17, 21, 22, 23, 27, 28, 29, 30, 31, 34, 42, 43 |
| Reduced treatment time | 17 | 1, 13, 14, 15, 17, 21, 22, 23, 27, 28, 29, 30, 31, 34, 42, 43 |
| **Professional development** | | |
| New knowledge and competence | 29 | 1, 2, 3, 6, 11, 12, 13, 14, 15, 16, 17, 18, 21, 22, 23, 24, 25, 26, 27, 28, 29, 30, 31, 32, 33, 34, 35, 42, 43 |
| Redevelopment of tasks and responsibilities | 23 | 13, 14, 15, 16, 17, 18, 21, 22, 23, 24, 25, 26, 27, 28, 29, 30, 31, 32, 33, 34, 35, 42, 43 |
| Increased interdisciplinary teamwork | 20 | 1, 2, 3, 6, 13, 14, 15, 16, 17, 18, 21, 22, 23, 24, 25, 26, 27, 28, 29, 30, 31, 32, 33, 34, 35 |
| Increased autonomy | 5 | 21, 22, 23, 24, 25 |
| **Hospital profiling** | | |
| Showing potential high-quality care | 30 | 2, 3, 4, 7, 8, 9, 13, 14, 15, 16, 17, 18, 19, 20, 21, 22, 23, 24, 27, 28, 29, 30, 31, 33, 34, 35, 36, 38, 40, 41 |
| Hospital differentiation | 29 | 2, 3, 6, 7, 8, 9, 13, 14, 15, 18, 19, 20, 22, 23, 24, 27, 28, 29, 30, 31, 33, 34, 35, 36, 38, 40, 41, 42, 43 |
| Potential increase in patient demand | 23 | 2, 3, 7, 13, 14, 15, 16, 17, 19, 20, 21, 22, 23, 27, 28, 30, 31, 35, 36, 38, 39, 40, 41 |

Appendix C: Frequency of respondents who discussed barriers to the implementation of MR-Linac into prostate cancer care, by main theme and subtheme.

| **Barriers** | **Total** | **Respondent ID, following Table 1** |
| --- | --- | --- |
| **Technical complexities** | | |
| New requirements in knowledge and competence | 29 | 1, 3, 6, 11, 12, 13, 14, 15, 16, 17, 18, 19, 21, 22, 23, 24, 25, 26, 27, 28, 29, 30, 31, 32, 33, 34, 35, 42, 43 |
| Continuous software development | 22 | 1, 13, 14, 15, 16, 17, 18, 21, 22, 23, 24, 25, 27, 28, 29, 30, 31, 33, 34, 35, 42, 43 |
| Absence of the conventional stability | 21 | 1, 13, 14, 15, 17, 18, 21, 22, 23, 24, 25, 27, 28, 29, 30, 31, 33, 34, 35, 42, 43 |
| Continuous learning curve | 20 | 13, 14, 15, 16, 17, 21, 22, 23, 24, 25, 27, 28, 29, 30, 31, 33, 34, 35, 42, 43 |
| **Substantial staffing and structural investments** | | |
| Changing tasks and responsibilities | 28 | 2, 3, 6, 7, 8, 9, 13, 14, 15, 16, 17, 19, 20, 21, 22, 23, 24, 27, 28, 29, 30, 31, 32, 33, 34, 35, 42, 43 |
| Additional operational requirements | 26 | 2, 3, 4, 5, 6, 7, 8, 9, 13, 14, 15, 16, 17, 20, 21, 27, 28, 29, 30, 31, 32, 33, 34, 35, 42, 43 |
| Training and development programs | 25 | 2, 3, 6, 7, 13, 14, 15, 16, 17, 21, 22, 23, 24, 25, 27, 28, 29, 30, 31, 32, 33, 34, 35, 42, 43 |
| **Lack of empirical evidence of clinical benefits** | | |
| Uncertain clinical benefit | 28 | 2, 3, 5, 6, 7, 9, 12, 13, 14, 15, 18, 19, 20, 21, 22, 27, 28, 30, 31, 32, 33, 35, 36, 40, 41, 42, 43 |
| Ethical issues | 18 | 3, 7, 8, 9, 11,12, 13, 14, 15, 16, 18, 19, 20, 27, 30, 31, 40, 41 |
| Uncertain return of investment | 13 | 7, 15, 18, 20, 22, 23, 24, 25, 28, 30, 32, 33, 35 |
| **Professional silos** | | |
| Conservative culture | 31 | 2, 3, 4, 6, 7, 8, 9, 11, 13, 14, 15, 18, 19, 20, 21, 22, 23, 24, 25, 26, 27, 28, 29, 30, 31, 33, 34, 35, 36, 38, 42 |
| Silo mentality | 26 | 2, 3, 6, 7, 9, 14, 15, 18, 19, 20, 22, 23, 24, 27, 28, 29, 31, 34, 35, 36, 38, 39, 40, 41, 42, 43 |
| Publication pressure | 12 | 7, 10, 12, 14, 18, 19, 28, 32, 34, 38, 39, 41 |
| **Patient referral patterns** | | |
| Competitive specialties | 30 | 2, 3, 6, 7, 8, 13, 14, 15, 16, 17, 18, 19, 20, 21, 27, 28, 29, 30, 31, 32, 33, 35, 36, 37, 38, 39, 40, 41, 42, 43 |
| Competitive hospitals | 28 | 2, 3, 6, 7, 10, 11, 13, 14, 15, 16, 18, 19, 20, 21, 28, 29, 30, 31, 32, 33, 34, 35, 36, 37, 40, 41, 42, 43 |
